# Treatment of Intermittent Fever by Quinidine

**Published:** 1854-09

**Authors:** William Pepper

**Affiliations:** Physician to the Pennsylvania Hospital


*Treatment of Intermittent Fever by Quinidine.* By WILLIAM PEPPER, M. D., Physician to the Pennsylvania Hospital.

In January, 1853, I published in the Amer. Jour. of Med. Sciences, a number of cases of intermittent fever, treated by the sulphate of cinchonia. From that time up to the present, I have used no other remedy in my hospital practice for this class of diseases, and the result has fully confirmed the conclusions then obtained. In April last, at the suggestion of Dr. Conrad, I was induced to try the recently discovered alkaloid, quinidine, in place of the cinchonia; unfortunately, however, the number of suitable cases of intermittent, admitted during the past spring, was unusually limited, amounting in all to only five cases, and entirely too few to establish any positive conclusion in therapeutics, and yet sufficient to invite the attention of the profession to a more extended trial of its virtues.

Before proceeding to report these cases, it may be well to state a few facts in regard to the remedy under consideration. As far back as 1833, Henry and Delondre discovered this alkaloid, and gave it its present name; the following year, however, further investigations induced them to believe that it was identical with quinine. In 1848, Winckler, a distinguished German chemist, gave a full description of it, and some of its salts; he at first, was also disposed to consider it as a mere hydrate of quinine; but by patient investigation, finally proved that it was a distinct substance, possessed of many distinct physical and chemical properties. In the last edition of Pereira's Materia Medica and Therapeutics for 1854, it is stated that the quinidine may be obtained from most of the genuine cinchona barks, by the same processes that are used for procuring quinine; the sulphate of quinidine being more soluble than the same salt of quinine, the former is left in the mother waters. The most important fact, however, in connection with this subject is, that this new alkaloid is found to abound in the cheaper kinds of barks from New Grenada; the Bogota cinchona, which contains but little quinine and a large amount of quinidine, is now largely used in England for obtaining this last named alkaloid. It can now be obtained at Powers & Weightman's, Manufacturing Chemists of our city, at fifty cents an ounce less than quinine; and there is no doubt that it could be supplied by the importation of the cheaper kinds of bark, at a price not exceeding that of cinchonia itself.

The sulphate of quinidine used at the hospital, presented much the same appearance as this salt of quinine, nor was there any marked difference in their taste or solubility. For those who desire to investigate more fully, the chemical and physical properties of this substance, I would refer to an elaborate paper by Winckler, in the Pharmaceutical Journal for 1848, vol. vii, p. 527; as also to an article by the same author, in Chemical

Gazette, vol. vi, p. 164. No allusion, however, is here made to its therapeutic properties, nor have I been able to refer to any author, where such information might be obtained. Mr. Procter, Ed. Journal of Pharmacy, informed me that he had met with the same difficulty, and that with the view of elucidating this all important point, he had quite recently requested a medical friend to test its powers in intermittent fever; the result, he states, was most satisfactory in the only four cases of obstinate disease in which it was tried. In reporting the following five additional cases, I shall be as brief as possible.

Case 1st.—A laborer, æt. 26 years, entered the hospital April 5th, with intermittent fever of the tertian form, the paroxysm generally coming on at 10 A. M.; he had been suffering with the disease for two weeks. On the 6th, he had a severe chill, lasting thirty minutes, and followed by the usual hot and sweating stages. Accordingly on the morning of the 8th, he was directed quinidine sulph. grs. x, grs. ij. every hour, commencing at 5 o'clock.

From this time the patient had no return of the disease, and although he remained in the institution until the middle of June, had no relapse during that time.

Case 2d.—T. W., laborer, admitted May 27th, stated that he had been subject to chills and fever for four months, and that he had contracted the disease in Savannah. At first, the paroxysms appeared to have come on about 10 o'clock, every other day; lately, however, they had assumed the quotidian type, but still occurred at the same period of the day.

Although the patient had a chill on the day of his admission, as also on the following day, no treatment was instituted until the 29th, when he took the quinidine sulph. grs. ij. every hour, commencing at 8 A. M., or just five hours before the expected paroxysm. The only perceptible effect in this instance, however, was the mitigation of the chill, and its postponement for about one hour. On the following day, the same treatment was assumed, and with the most perfect success; he remained in the house about ten days after this, but during his stay had no relapse.

Case 3d.—A young Irish woman, aged 22 years, was admitted June 2d.; she stated that she had been suffering all last Fall with chills and fever, and that she was finally cured of her disease, after entering this hospital. Since then, she has remained perfectly well up to May 20th, when the chills again appeared, and continued to recur daily at about 10 A. M.

With the view of ascertaining the character of the attacks, it was deemed most expedient not to interfere until the 5th, or three days after admission. She now took quinidine sulph. grs. ij. every hour, commencing at 5 A. M., until in all grs. x, had been taken; without, however, checking the paroxysm, though it was certainly considerably mitigated.

On the following day, June 6th, the same plan was pursued, and with the effect of completely checking the disease; which, in fact, did not return during the ten days she remained in the hospital.

CASE 4th.—A laborer, aged 48 years, entered the house June 13th, suffering with a severe chill; he had the same on the five previous days, the paroxysms coming on in the course of the forenoon, but rather irregularly. On the 14th, he had a chill, lasting nearly two hours, and commencing about 10 o'clock, A. M.; on the following day, he took the quinidine grs. x, as in the former instance, grs. ij, every hour in anticipation of the chill. It, however, came on at the usual time, but was exceedingly mild and lasted only thirty minutes.

On the 16th, the remedy was administered in like manner; upon this occasion it effectually checked the disease, nor was there any relapse up to July 1st, when he left the Institution. In this case, it should be mentioned, that as the patient was somewhat anæmic, and had enlargement of the spleen, gr. i. of the quiuidine combined with gr. v. of carb. Ferri, (Vallet,) was continued three times a day, during his stay in the house.

CASE 5th.—A sailor from Mobile, aged 22, entered June 17th, sick two weeks with intermittent fever; the attacks coming on daily at about mid-day. This patient was somewhat prostrate and slightly jaundiced.

The treatment by quinidine was commenced on the 19th; the paroxysms, however, came on one hour earlier than upon the previous day, and the chill was quite as severe as usual. On the 20th, he again took grs. ij, for five consecutive hours in anticipation of a return; but from this time until he left the hospital, June 27th, he remained perfectly well.

Excepting in the first case above reported, the remedy had to be repeated a second time, before the fever could be fully arrested, but in no instance was its further use necessary. It is to be regretted that the cases could not have been retained longer under observation, so as to have fully ascertained as to the permanency of the cure; but the crowded state of the wards, and the unwillingness of the patients to remain when they felt perfectly well, rendered it imposible to effect this desirable end. It will be perceived that grs. x was the largest amount given upon any one day; and as I have generally, under apparently similar circumstances, been obliged to give grs. xv. of sulph. quinia or cinchona, I am disposed to believe that the quinidine is more active than either of these alkaloids. In England it is manufactured chiefly with the view of adulterating quinine; but if the above conclusion be confirmed by further observation, our patients' health will not suffer by such admixture, however, unfavorably it may operate upon them in a pecuniary point of view.

In conclusion, I would remark that Dr. Darrach, to whom I am much indebted for notes on the above cases, is anxious to continue the use of this remedy in all suitable cases that may occur during his residence in the hospital, so as to enable him, at some future period, to make a more ample report upon this subject.

### *Ovarian cyst containing pus and gas.*

A young married woman, aged 23 years, entered the hospital December 26th, suffering with pain and distention of the abdomen. She stated that about eighteen months ago, after exposure to cold, she had been suddenly seized with severe pain in the back and lower part of the belly, two weeks from which time she first perceived the swelling, but could not state whether it commenced on the right or left side. Before admission into the hospital she had been twice tapped, a large amount of fluid being drawn off at each operation; the catamenia had ceased about one year.

She came under my care March 1st, and then presented the following symptoms. Constant hectic fever, bowels obstinately constipated, total loss of appetite, and extreme emaciation. The abdomen was enormously swollen, measuring fifty-three inches in circumference, and causing great oppression by the upward pressure of the diaphragm ; the superficial veins were also much enlarged and distended with dark blood. The most remarkable phenomenon in the case, however, was the exceeding great resonance of percussion over the entire swelling, the slightest tap eliciting a peculiar metallic ringing sound, such as I never recollect to have heard in ordinary cases of tympanitis. The patient had been freely purged with elaterium, and had also used an infusion of juniper berries with cream of tartar. By the 1st of March this plan of treatment was abandoned, and she was directed olei terebinth gtt. xx. in emulsion four or five times a day ; and the same remedy was occasionally administered by enema, with the view of removing the supposed tympanitic distention and constipation of the bowels. In the mean time her strength was sustained by wine whey, beef essence, and other supporting means ; but despite these measures she gradually sank, and expired on the 16th of March.

Examination post mortem, on the following day, presented nothing remarkable in the thoracic organs excepting what might be fairly attributable to the upward pressure of the diaphragm ; in like manner the contents of the abdomen, with the exception of the left ovarium, were in a healthy condition. This latter, however, was converted into a vast cyst, adhering throughout in front to the abdominal parietes, forcing the stomach upwards and backwards, and completely concealing the small intestines. Upon puncturing the sack there was an audible escape of fetid gas, followed by a slight subsidence of the swelling ; a large incision gave vent to about ten gallons of offensive pus, mixed with shreds of semi decomposed false membrane, and the entire inner surface of the cyst was evidently in a sphacelated condition.

The above case goes to show that under certain circumstances we may have a tympanitic resonance on percussion over an avarian cyst, quite independent of any flatus contained in the alimentary canal. In chronic pleurisy or empyema it is not uncommon to find sulphuretted hydrogen evolved into the pleural sac, and giving rise to tympanitic percussion ; but such an occurrence in case of an inflamed ovarian cyst has not before fallen under my observation. The fact is important, since such a state of things might lead to an erroneous diagnosis in cases which had not been long under treatment, or where no perfect history could be obtained.

